# Expanded phenotypes and pathogenesis of geleophysic dysplasia 3 resulted from a de novo LTBP3 mutation: A case report

**DOI:** 10.1097/MD.0000000000041000

**Published:** 2024-12-20

**Authors:** Jie Liang, Yu Han, Huimin Tao, Xuezhen Wang, Bei Zhang, Jiebin Wu, Jingfang Zhai

**Affiliations:** a Xuzhou Clinical College of Xuzhou Medical University, Xuzhou Central Hospital, Xuzhou, China; b Department of Prenatal Diagnosis Medical Center, Xuzhou Central Hospital, Xuzhou, Jiangsu, China.

**Keywords:** expanded phenotypes, geleophysic dysplasia 3 (GPHYSD3), latent TGF-β binding protein-3/LTBP3, pathogenesis, transforming growth factor β (TGF-β)

## Abstract

**Rationale:**

The aim of this study is to investigate the de novo mutation and clinical features of latent transforming growth factor-beta-binding protein 3 (LTBP3) gene-associated geleophysic dysplasia 3, and possible mechanisms of action.

**Patient concerns:**

A nonconsanguineous couple was recruited for this study due to the presence of intrauterine growth restriction. The pregnant woman and her elder daughter presented with skeletal abnormalities with diabetes. The pregnant woman underwent amniocentesis for cytogenetic analysis and copy number variation sequencing. Furthermore, we employed a combination of pedigree whole exome sequencing and bioinformatics analysis to predict the effects of mutations.

**Diagnoses:**

The results of karyotyping and copy number variation sequencing were normal. And the whole exome sequencing results indicated that the family carried a de novo mutation c.852_853insAGG (p.L284_P285insR) in the LTBP3 gene (NM_001130144.3) inherited from the mother. The results of bioinformatics prediction demonstrated the mutation influenced the stability of the LTBP3 gene, thereby enhanced the transforming growth factor β signaling pathways.

**Interventions:**

The couple terminated the pregnancy after comprehensive consideration.

**Outcomes:**

A de novo non-frameshift mutation of the LTBP3 gene might enhance the transforming growth factor β signaling pathways, thereby leading to geleophysic dysplasia 3.

**Lessons:**

As a rare multi-system musculoskeletal disorder, geleophysic dysplasia 3 necessitates early prenatal diagnosis and multidisciplinary consultation in order to facilitate further diagnosis and evaluation of the patient and the fetus.

## 1. Introduction

Geleophysic dysplasia 3 (GPHYSD3), as an autosomal dominant genetic disease, is typically characterized by short stature, brachydactyly, progressive joint limitation and contractures, distinctive facial features, progressive cardiac valvular disease, and thickened skin,^[1]^ especially at birth or even in utero. Since the initial report with the latent transforming growth factor β (TGF-β) binding protein-3 (LTBP3) gene in 2009,^[2]^ only 2 cases associated with LTBP3-GPHYSD3 mutations have been documented.^[1]^

The LTBP3 gene (OMIM 602,090) on chromosome 11 is composed of 13 epidermal growth factor-like repeats and 4 TGF-β binding (TB) domains (or 8 cysteine domains), as well as insulin-like growth factor binding protein domains.^[1,3]^ Moreover, patients with GPHYSD exhibited the enhanced TGF-β signaling.^[4]^ The bioavailability of TGF-β is regulated by controlling itself through itself secretion, folding, activation, and deposition into the extracellular matrix, involving multiple systems.^[5]^ Therefore, GPHYSD3 is closely associated with either the mutation sites of the LTBP3 gene or the changes of TGF-β signaling pathways. In this report, the identification of a heterozygous non-frameshift mutation (NM_001130144.3: exon 3: c.852_853insAGG: p.L284_P285insR) in the TB domain of the LTBP3 gene was observed in a dominant GPHYSD3 family.

Apart from the fetus consistently exhibiting shortened limbs, similar with both the pregnant woman and her elder daughter, both of the latter additionally displayed abbreviated hands and feet, thickened skin on hands and feet, especially expanded phenotype-type 1 diabetes unreported previously associated with the LTBP3 gene. In the study, based on the clinical manifestations of GPHYSD3 and related literature, a comprehensive possible pathogenesis between the phenotype and genotype of LTBP3-GPHYSD3 has been identified.

## 2. Materials and methods

The study was approved by the ethics committee of Xuzhou Central Hospital (XZXY-LK-20230314-035). The participants provided their written informed consent in this study.

### 2.1. Ultrasound examination

Prenatal ultrasound examination was conducted by ultrasound physicians using a color Doppler ultrasound instrument (GE Voluson E8, American) in accordance with the guidelines of the International Society of Ultrasound in Obstetrics and Gynecology. The fetus was diagnosed with intrauterine growth restriction.^[6,7]^

### 2.2. Karyotyping

Amniotic fluid was collected via ultrasound-guided amniocentesis, and the collected cells were subsequently cultured, harvested, prepared, and subjected to G-banding. Karyotype report was interpreted in accordance with the International System for Human Cytogenetic Nomenclature (ISCN 2016/ISCN 2020).

### 2.3. Copy number variation sequencing

Fetal genomic DNA and DNA libraries were constructed in accordance with the instructions by the manufacturer on the fetal samples obtained above, and then subjected to PCR amplification and sequencing. Subsequently, the NextSeq550AR platform was employed for large-scale parallel sequencing. The sequencing data were then compared with the human reference genome sequence (GRCh38/hg38) and the corresponding result was carried.

### 2.4. Whole exome sequencing

Genomic DNA of obtained fetal samples and parental peripheral blood were captured, hybridized and enriched by using SureSelect Human All Exon kit v6 (Agilent Technologies). The raw data was obtained by double-ended sequencing using the Illumina Novaseq6000 platform (Illumina, San Diego, CA). The obtained sequences were compared with the GRCH38 reference genome using BWA software, followed by duplicated sequences removed by PCR, and base quality correction performed by Genome Anlysis Toolkit (Version 3.8.0.0). The Genome Anlysis Toolkit tool was employed to identify single nucleotide polymorphisms and insertion/deletion in the samples and to filter them. Subsequently, the filtered variations were subjected to annotation, interpretation and functional verification. All variants were analyzed according to the American College of Medical Genetics.^[3]^

### 2.5. Bioinformatics analysis

STING database (https://cn.string-db.org/) was used to predict the correlations between proteins. Additionally, we completed structural modeling simulation to predict the structure of both the wild-type and mutant LTBP3 by AlphaFold2 website (https://colab.research.google.com/github/sokrypton/ColabFold/blob/main/AlphaFold2.ipynb). The resulting protein structures were subsequently subjected to visual analysis using Pymol software (The PyMOL Molecular Graphics System, Version 1.8.4.0). And the Figdraw (https://www.figdraw.com/#/) was used to map the signaling pathways mechanism.

## 3. Case presentation

**Figure 1.**
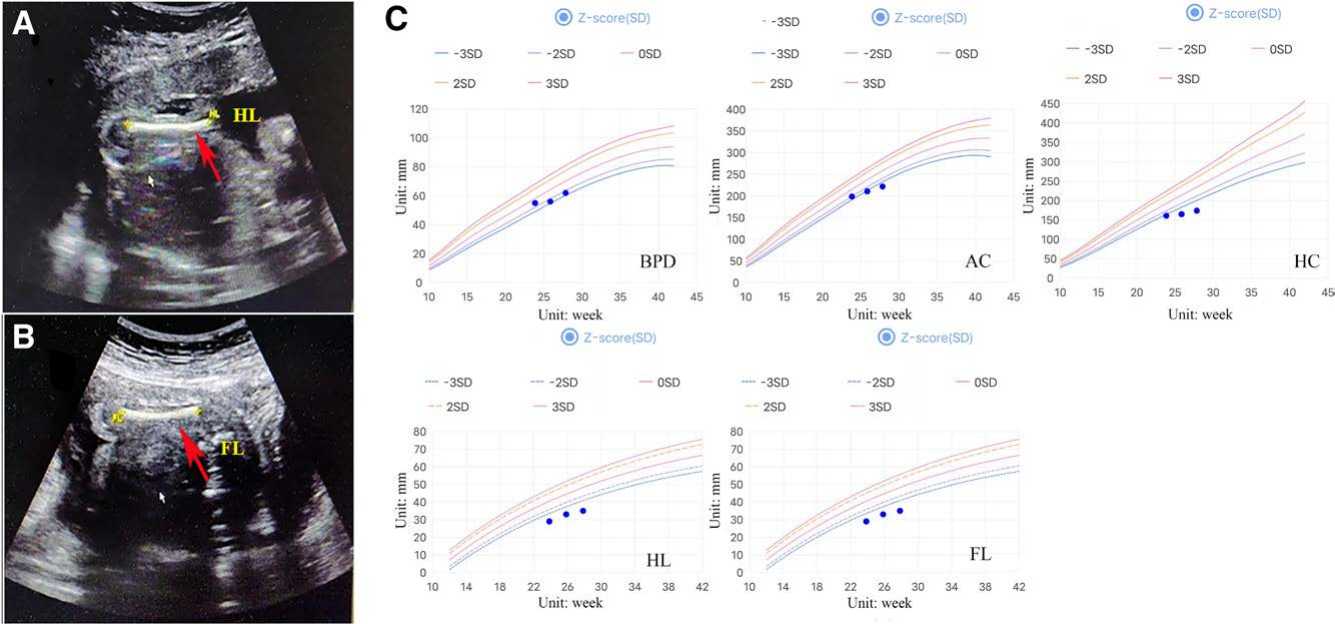
(A) Fetal femur length (FL); (B) fetal humerus length (HL); and (C) fetal growth curves of biparietal diameter (BPD), head circumference (HC), and abdominal circumference (AC), femur length (FL), humerus length (HL) at 23 + 6, 25 + 6, and 27 + 6 weeks.

**Figure 2.**
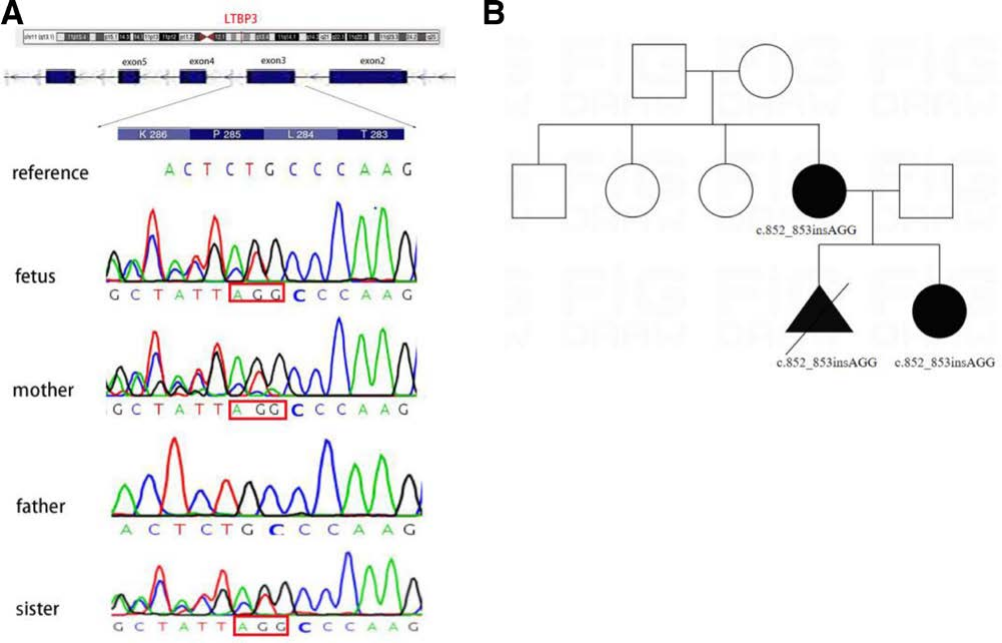
(A) The non-frameshift variants of LTBP3 gene on chromosome 11q23.3, c.852_853insAGG (p.L284_P285insR) in exon 3 inherited from the mother. (B) Pedigree of family with GPHYSD3.

A 34-year-old pregnant woman (gravida 5, para 2) was transferred to prenatal diagnosis medical center of Xuzhou Central Hospital in November 2021 for high risk of trisomy 21 and trisomy 18 at 13 + 1 weeks of gestation. At the following 23 + 6 weeks, ultrasound revealed fetal relatively short-limb morphology: femur length 28 mm (−7 standard deviation [SD]), humerus length 29 mm (−5SD). Dynamic ultrasound at the following 25 + 6 weeks of gestation still indicated fetal short limbs: femur length 32 mm (−7.1SD), humerus length 33 mm (−4.8SD) (Fig. 1A, B), suggesting intrauterine growth restriction. After a duration of 2 weeks, the ultrasound revealed worsen short bone length in all 4 extremities: femur length 34 mm (−8SD), humerus length 35 mm (−5.4SD) (Fig. 1C). The results of karyotyping and copy number variation sequencing were normal. And trio-whole exome sequencing was performed in this family, the fetus and the eldest daughter was identified with a de novo heterozygous variant of the LTBP3 (NM_0011301443) gene, c.852_853insAGG (p.L284_P285insR) in exon 3 inherited from the mother, which resulted in a non-frameshift insertion (Fig. 2). The couple terminated the pregnancy after comprehensive consideration. After reexamining her history, she once had a history of intrauterine fetal death at full term in 2009 and 2 miscarriages, but the precise reasons remained undetermined. However, she denied drug usage. Neither the couple nor the grandparents were consanguineous.

**Figure 3.**
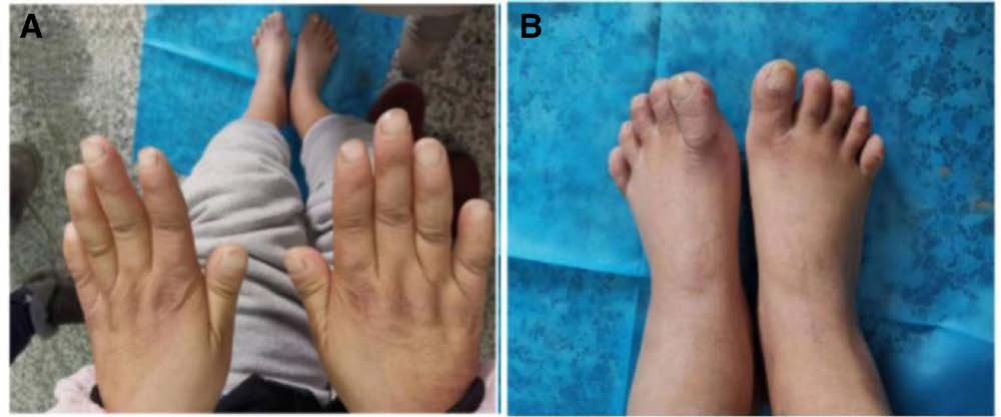
(A) mother’s short hands; (B) mother’s short feet.

The pregnant woman herself was short, 150 cm tall, with short hands and feet, thickened skin on hands and feet (Fig. 3A, B), moderate anemia, history of hypertension and hypothyroidism and type 1 diabetes and diabetic nephropathy (DN) under control. In addition, she experienced shortness of breath and dyspnea due to tracheal stenosis and severe lung infection in 2021. An ultrasound in January 2021 revealed mild instances of mitral and tricuspid regurgitation, left atrial enlargement and pulmonary hypertension. Furthermore, the woman had a family history of type 1 diabetes. Moreover, the mother of a pregnant woman also suffered from type 1 diabetes. The woman’s 10-year-old elder daughter, 123 cm tall (−2SD), had type 1 diabetes and exhibited an unreported speech impediment.

## 4. Discussion

**Figure 4.**
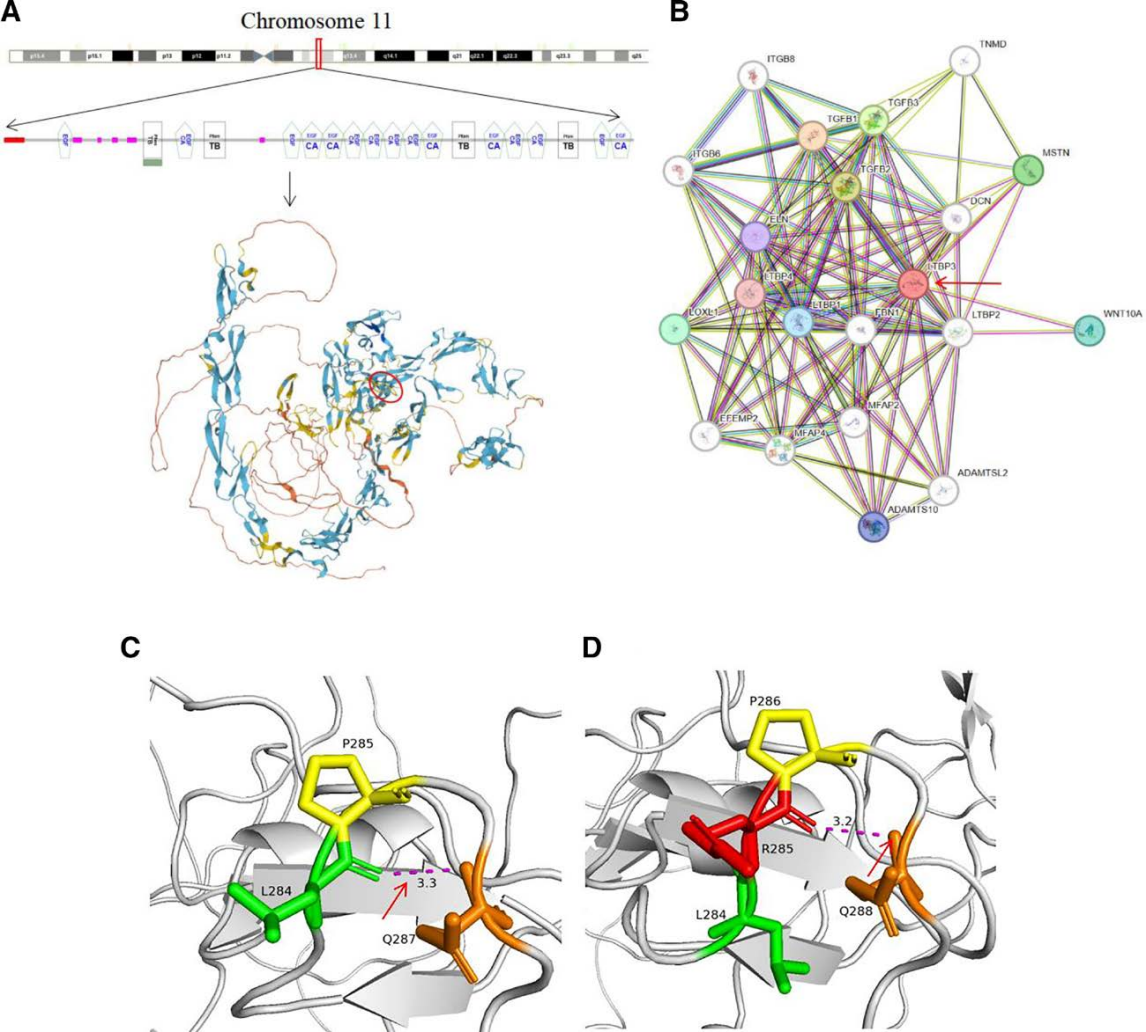
(A) The chromosome location, structural sequence and protein structure prediction of LTBP3 gene on the top, three-dimensional structure model of wild-type LTBP3 gene, and c.852_853insAGG mutation resulted in the change of the local region in the red circle. (B) The analysis of protein–protein interaction network showed that LTBP3 gene is the key gene in the protein interaction network. (C) Wild type LTBP3 gene protein prediction map, 284 leucine and 287 glutamine hydrogen bond link. (D) Protein prediction map of mutant LTBP3 gene with hydrogen bond link between 285 arginine and 288 glutamine. LTBP3 = latent transforming growth factor-beta-binding protein 3.

The rare GPHYSD3 has been confirmed primarily to lead to a de novo mutation of LTBP3 gene.^[8]^ The de novo non-frameshift mutation (NM_001130144.3: exon 3: c.852_853insAGG: p.L284_P285insR) of the LTBP3 gene has not been reported in the dominant GPHYSD3 family of our study up to now. This LTBP3 gene has been confirmed to widely express in various tissues including bones, teeth, heart, and aorta.^[5]^ Furthermore, LTBP3 gene is the key gene in the protein interaction network (Fig. 4B). The mutation of the LTBP3 gene results in structural and functional changes of the related proteins, leading to the development of 3 distinct disorders: acromicric dysplasia (OMIM 102,370), dental abnormalities and short stature (OMIM 601,216), and gelephysical dysplasia 3 (GPHYSD3) (OMIM 617,809).^[8–10]^ The above mutation explained our patients’ range of clinical manifestations, all of whom presented with skeletal abnormalities, fetal limb shortening, maternal and elder daughter’s short stature, as well as shortened hands and fingers. Based on the patient-specific clinical manifestations and gene-targeted testing, the diagnosis of GPHYSD3 was clear.

However, the occurrence of mild mitral and tricuspid regurgitation, left atrial dilatation, pulmonary hypertension, type 1 diabetes and DN in pregnant woman, as well as in the elder daughter with speech difficulties, have not been previously documented. Notably, no cardiac valvular involvement was found in our 3 patients, compared to the clinical characteristics of LTBP3-GPHYSD3 published in Table 1. Above the features, along with hepatomegaly and premature mortality, may serve as the primary criteria for distinguishing GPHYSD3 from Weill-Marchesani syndrome, acromicric dysplasia, and Myhre syndrome.

**Table 1:**
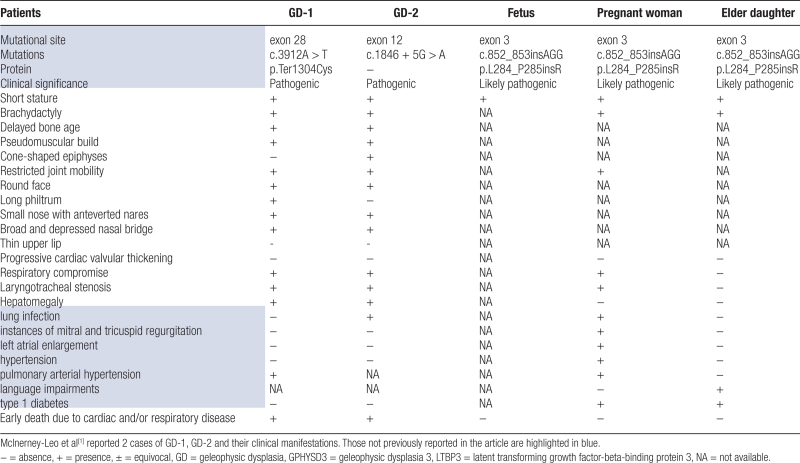
Published reports with LTBP3-GPHYSD3.

The c.852_853insAGG: p.L284_P285insR mutation in the TB domain of the LTBP3 gene (PM1) changes an altered protein length without affecting the open reading frame (PM4) (Fig. 4A). All of our patients presented with similar phenotype due to the same variant, which not only segregated with the disease in this family (PP1), but also conformed to the LTBP3 single-gene inheritance pattern (PP4). And this mutation was not revealed in genome aggregation database or other large sequenced populations (PM2). In total, the c.852_853insAGG: p.L284_P285insR mutation of LTBP3 gene should be classified to “pathogenic” type (PM1 + PM2 + PM4 + PP1 + PP4).

**Figure 5.**
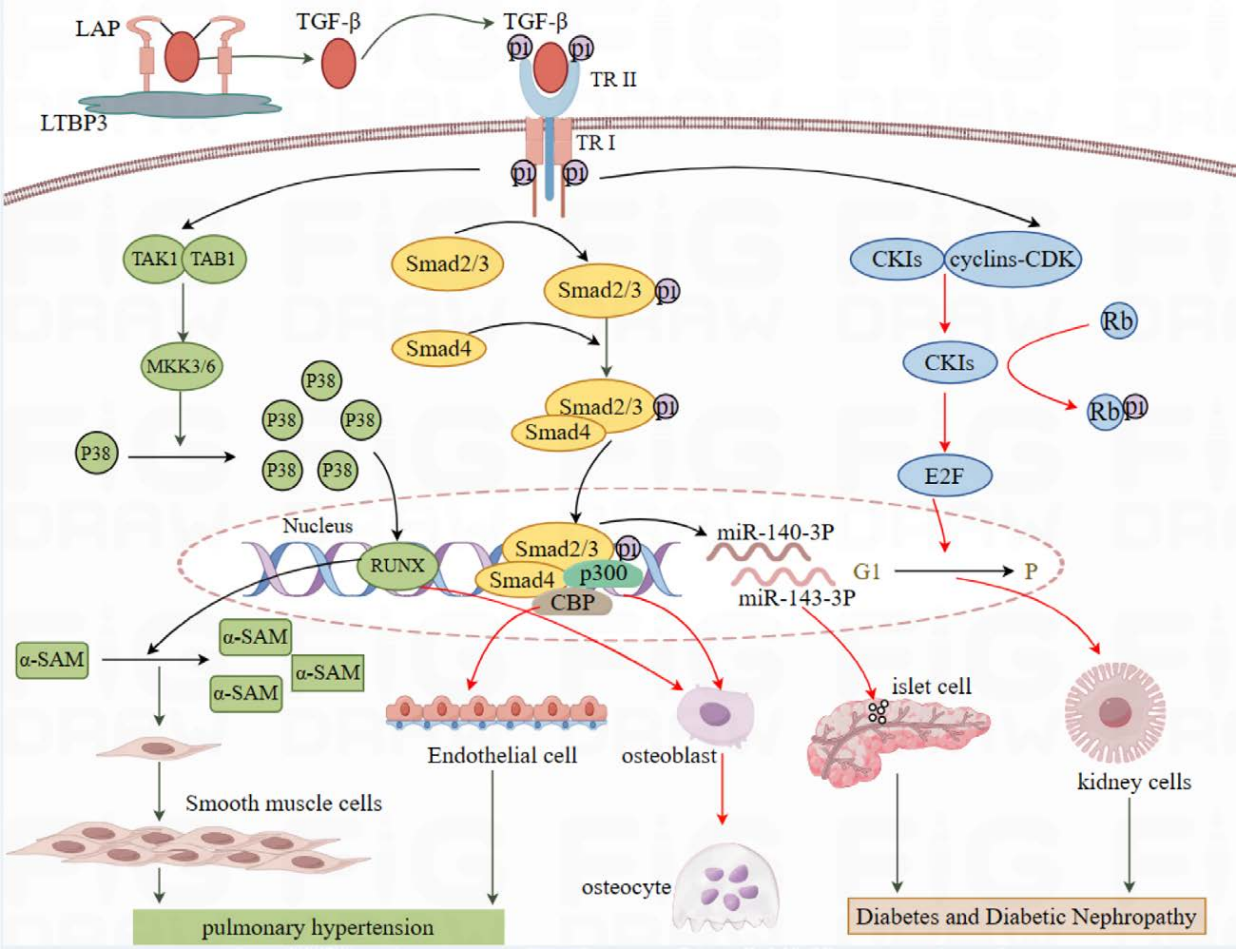
LTBP3 combined with SLC (TGF-β and LAP via disulfide bonds) significantly strengthen the bioavailability of TGF-β. The released TGF-β triggers both intracellular Smad-dependent and non-Smad-dependent signaling pathways. The pathway in yellow indicates Smad-dependent signaling pathway inhibiting the differentiation of osteoblasts, endothelial cells and islet cells. The p38/MAPK pathway in green not only exerts its effects on osteoblasts, but also promotes the proliferation and differentiation of smooth muscle cells. The last pathway in blue is that TGF-β causes hypertrophy of kidney cells. LAP = latency-associated protein, LTBP3 = latent transforming growth factor-beta-binding protein 3, SLC = small latent complex.

Meanwhile, the wild-type LTBP3 gene uses a hydrogen bond between leucine (284) and glutamine (287). However, the insertion of arginine (AGG) between leucine (284) and proline (285) resulted in a structural alteration of the LTBP3 protein due to the changes of the hydrogen bond between the amino acids, and the stability of LTBP3 combined with TGF-β was decreased (Fig. 4C, D), which ultimately resulted in the release of TGF-β increasingly, thereby enhanced the signaling pathways. Moreover, the enhancement of TGF-β signaling has been previously reported in GPHYSD patients with in the TB domain of the LTBP3 gene.^[4]^ Therefore, the corresponding mechanism pathways among the LTBP3 gene, TGF-β signaling pathways and GPHYSD3 were plotted in Figure 5. GPHYSD3 is primarily initiated by the TGF-β signaling pathways, where a small latent complex is formed of TGF-β and latency-associated protein via disulfide bonds.^[11]^ Furthermore, LTBP3 combined with small latent complex significantly updates the bioavailability of TGF-β.^[5]^ The connection of TGF-β type II receptor on the cell membrane leads to phosphorylation of the TGF-β type I receptor, which triggers intracellular Smad-dependent and non-Smad-dependent signaling pathways.^[8,12]^ The Smad-dependent pathway in yellow involves the activation of TGF-β type I receptor, then binds to Smad2 and Smad3, facilitating their self-phosphorylation and subsequent dissociation. Subsequently, the phosphorylated Smad2/3 complex binds with Smad4, translocates to the nucleus, and recruits co-factors for regulating target genes.^[8,13]^ The maturation and differentiation of osteoblasts are ultimately impeded by this pathway. Simultaneously, the Smad2/3 signaling pathway also impairs endothelial cell function and viability.

However, in the green non-Smad-independent pathway, the p38/MAPK pathway is regulated by TGF-β through the phosphorylation of TGF-β-activated kinase 1, leading to the recruitment of TGF-β-activated kinase 1-binding protein 1,^[14]^ further bolstering transcriptional activity of transcription factors. The signaling pathway not only exerts its effects on osteoblasts, but also upregulates the expression of a-smooth muscle actin and promotes the proliferation and differentiation of smooth muscle cells.^[8,15,16]^ Therefore, the abnormal regulations of these pathways not only results in skeletal system abnormalities, but also contributes to the development of pulmonary hypertension. In addition, the LTBP3-deficient mice display postnatal developmental delay and pulmonary insufficiency in GPHYSD.^[1]^ The above mentioned mechanism could account for the alterations in the skeletal system, cardiopulmonary disease.

Additionally, the presence of diabetes and DN in pregnant woman is linked to the pathway in blue. TGF-β can mediate RNA processing through the Smad2/3 pathway, activate the secretion of miR-140-3P and miR-143-3P,^[17]^ reduce the number and activity of islet cells, and ultimately mediate the apoptosis of islet cells, which can lead to type 1 diabetes. Meanwhile, the overactivation of the TGF-β signaling pathways results in an abnormal increase in the expression of cyclin-dependent kinase inhibitor, which leads to the competitive binding of cyclin-dependent kinase inhibitor with cyclin-dependent kinases in the complex.^[18]^ This inhibits cyclin-dependent kinases activity and the phosphorylation of the Rb protein, thereby preventing cells from entering the G1 phase. In turn, this causes renal cell hypertrophy, which is a contributing factor to the development of early DN.^[19]^ However, language barrier in the elder daughter remains unanalyzed and further clinical cases of the LTBP3 gene are required to validate the genetic association.

In summary, the study proposes a de novo variant of the LTBP3 gene that extends the phenotypes of GPHYSD3 disease and elucidates the mechanism of action of LTBP3-GPHYSD3. AS a rare multi-system musculoskeletal disorder, GPHYSD3 necessitates early prenatal diagnosis and multidisciplinary consultation in order to facilitate further diagnosis and evaluation of the patient and the fetus. Therefore, it is recommended for the women with GPHYSD3 to seek professional pregnancy assistance at a reproductive center prior to conception and to undergo a comprehensive prenatal diagnostic assessment.

## Acknowledgments

The authors thank the family for participating and supporting this study.

## Author contributions

**Conceptualization:** Jingfang Zhai, Jiebin Wu.

**Data curation:** Jie Liang, Yu Han, Xuezhen Wang, Huimin Tao.

**Formal analysis:** Jie Liang, Bei Zhang, Jiebin Wu.

**Funding acquisition:** Bei Zhang, Jingfang Zhai.

**Methodology:** Xuezhen Wang.

**Project administration:** Huimin Tao, Xuezhen Wang, Bei Zhang, Jiebin Wu, Jingfang Zhai.

**Software:** Jie Liang, Yu Han, Huimin Tao.

**Writing – original draft:** Jie Liang, Yu Han, Jingfang Zhai.

**Writing – review & editing:** Jingfang Zhai, Jiebin Wu.
